# Study on the Tensile Behavior of Helical Auxetic Yarns with Finite Element Method

**DOI:** 10.3390/ma16010122

**Published:** 2022-12-22

**Authors:** Sai Liu, Zhaoqun Du

**Affiliations:** 1 College of Textile Science and Engineering (International Institute of Silk), Zhejiang Sci-Tech University, Hangzhou 310018, China; 2 Engineering Research Center of Technical Textiles, Ministry of Education, Donghua University, Shanghai 201620, China

**Keywords:** helical auxetic yarns, tensile behavior, finite element model, Poisson’s ratio

## Abstract

Complex yarns with helical wrapping structure show auxetic effect under axial tension and a wide perspective application. Experimental results suggested that initial helical angle was one of the most important structural parameters. However, the experimental method was limited and could not effectively explain the deformation behavior or auxetic mechanism. A finite element model of the helical auxetic yarn was built and used to analyze the interactive relationship between the two components and the stress distribution mode. The effectiveness and accuracy of the model was first verified by comparing with the experimental results. The simulation results showed that the complex yarn with initial helical angle of 14.5° presented the maximum negative Poisson’s ratio of −2.5 under 5.0% axial strain. Both the contact property between the two components and the radial deformability of the elastic core filament were key factors of the auxetic property. When the contact surfaces were completely smooth and the friction coefficient μ was set to 0, the complex yarn presented non-auxetic behavior. When the Poisson’s ratio of the core filament was 0, the complex yarn showed greater auxetic effect. During the axial stretching, the tensile stress was mainly distributed in the wrap filament, which led to structural deformation and auxetic behavior. A pair of auxetic yarns showed pore effect and high expansion under axial strain. Thus, it may be necessary to consider new weaving structures and preparation methods to obtain the desired auxetic property and application of auxetic yarns.

## 1. Introduction

When stretched along one direction, auxetic materials [1] undergo an expanding deformation in the vertical direction. Structural design, preparation methods and property characterization of auxetic textiles have been reported [2]. Auxetic textiles present exceptional mechanical properties, especially synclastic curvature [3] and energy absorption [4], which are helpful for protective sports materials [5].

Auxetic yarns with helical wrapping structure were first reported to increase their diameter under axial tension [6]. Bhattacharya et al. [7] prepared helical auxetic yarn with a maximum negative Poisson’s ratio of −13.52. The effect of structural parameters [8], including diameter ratio, helical angle and tensile modulus of the wrap filament, on auxetic behavior was studied and analyzed. Complex yarns showed obvious auxetic behavior with higher diameter ratio, lower helical angle and larger tensile modulus of the wrap filament. Auxetic yarns with three components including coating structure [9] and braided structure [10] were also reported with negative Poisson’s ratio higher than −2 (i.e., less negative). The different mechanical properties and the interactive force between the yarn components were considered the main reasons for the auxetic effect. Hu et al. [11] prepared auxetic fabrics with three kinds of geometrical structures, foldable structure, rotating rectangle and reentrant hexagon, by a flat knitting method. Zhao et al. [12] manufactured auxetic warp knitted fabrics based on reentrant geometry with negative Poisson’s ratio of −0.5. Ng et al. [13] studied the open pore property and auxetic behavior of woven fabrics with auxetic yarns. The negative Poisson’s ratio of woven fabrics containing helical auxetic yarns was between −0.5 and −0.6 in [14], and the main structural parameters [15] included thread densities, weave design and yarn count. Cao et al. [16] reported bi-stretch woven fabrics with negative Poisson’s ratios of −0.36 and −0.27 when stretched along the warp and weft directions, respectively. All these results indicated that the negative Poisson’s ratios of auxetic fabrics were lower (i.e., less negative) than those of yarns, limiting their application. Thus, the deformation behavior of auxetic yarns and the interactive relationship between the components under axial strain first need to be verified, and the structures and methods for the production of auxetic fabrics woven with auxetic yarns have to be studied and improved.

Finite element method has also been widely used to simulate the mechanical properties and deformation behavior of textiles under tension, such as yarns [17], fabrics [18] and composites [19]. Wright et al. [20] presented the geometric model of a helical auxetic yarn, including the core and the wrap filament and stretching behavior, using finite element method. The contact condition of the two components of the yarn was set as tie constraint without sliding or friction effects. The effects of helical angle, diameter and modulus ratio on the Poisson’s ratio of the complex yarn were analyzed. The results showed that the complex yarn with smaller helix angle showed auxetic effect under lower axial strain. The diameter should be taken into consideration when selecting the wrap component with larger tensile modulus. Finite element analyses of helical auxetic yarns carried out by McAfee et al. [21], Du et al. [22] and Gao et al. [23] presented similar results with simulation software. Liu et al. [24] evaluated the effect of the elastic and hyperelastic core filament on the auxetic behavior. Additionally, a geometrical model [25], mechanical model [26] and artificial neural network and full factorial method [27] were also developed to predict the Poisson’s ratios of auxetic yarns. However, the unclear mechanism and the interactive relationship between the components should be studied and analyzed more deeply. 

Most of the results reported above were mainly regarding the effect of structural parameters on auxetic yarns. The key reason for auxetic behavior remains to be explained clearly. This study focused on the deformation behavior and auxetic mechanism. First, as a result of the most significant effect, systematic research about the helical angle is presented and was verified by experimental results. Second, the interactive relationship between the two components was analyzed in terms of the effect of the contact property (by friction coefficient) and the deformation property (by Poisson’s ratio). Then, the stretching behavior and auxetic effect of groups of auxetic yarns laid together was modeled for the simulated applicability of the auxetic textiles. Finally, the changing trajectory of centerline and stress distribution of the complex yarn were collected in an attempt to determine the auxetic mechanism.

## 2. Materials and Methods

**Figure 1:**
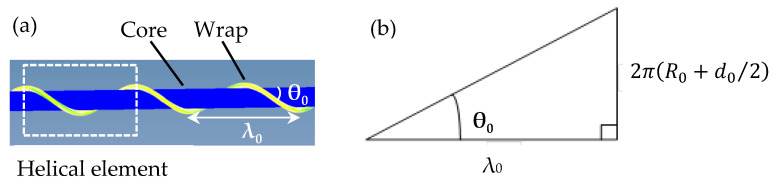
Structure of helical auxetic yarn: (**a**) helical wrapping structure; (**b**) expanded graph.

The complex yarn sample was prepared with nylon filament (fineness: 16.7 tex, Haoting fiber Co., Shaoxing, China) and polyurethane (fineness: 277.5 tex, Haoting fiber Co., Shaoxing, China) by a hollow spindle spinning machine (Wuxi No.7 Textile Machinery Co., Ltd, Wuxi, China). Polyurethane as the core was wrapped by nylon filament unwound from a bobbin. The spinning speed was set to 150 m/min. The finite element model of the complex yarn was built in ABAQUS software under the SI/mm unit system. A cylinder and a helix were assembled to be the geometric model of the auxetic yarn, with the assumption of uniformly circular cross sections. The auxetic structure and helical element are shown in Figure 1a, and λ_0_ was used to represent the length of a unit. The initial helical angle θ_0_ was one of the most important structural parameters, and it was defined as the angle between tangent direction of the wrap filament and horizontal line. 

The expanded structure of the complex yarn is shown in Figure 1b. *R*_0_ and *d*_0_ are the initial diameter of the core and the wrap filament without stretching. Then, λ_0_ could be calculated as follows:

λ_0_ =2π(R_0_+d_0_/2)/tanθ_0_ (1)

**Figure 2:**
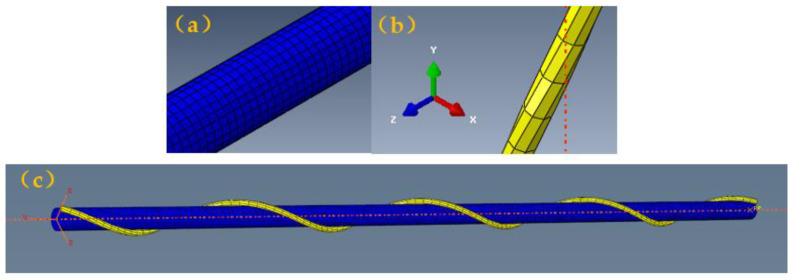
The meshes for the helical auxetic yarn: (**a**) core filament; (**b**) wrap filament; (**c**) complex yarn.

All six degrees of freedom of one end were set as zero in the initial step, and a uniaxial stretching speed load was applied to the other end in the analytical step. The boundary condition was applied to the complex yarn model selected with the geometrical structure and parameters above. Figure 2 shows the meshing of the complex yarn. The mesh type of the core filament is C3D8H with unit size of 0.1 mm^2^, and the mesh type of the wrapped yarn is C3D8 with unit size of 0.3 mm^2^. The deformation behavior of the complex yarn under axial strain was evaluated with the simulation and analytical process without damage criterion. 

## 3. Results

### 3.1. Poisson’s Ratios of Complex Yarns

#### 3.1.1. Finite Element Model Validation

**Figure 3:**
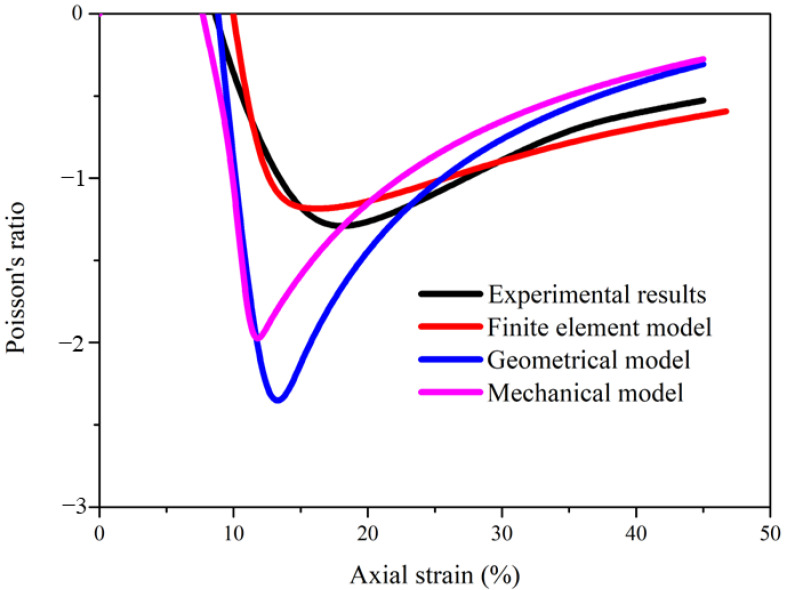
Comparison of Poisson’s ratio between experimental results and theoretical results.

The finite element model of the helical auxetic yarn was first verified on the basis of experimental and theoretical results. As shown in Figure 3, the variation trend of Poisson’s ratio by finite element simulation was consistent with experimental results of complex yarns with the same initial helical angle (26°). Compared with the results obtained by geometrical model [26] and mechanical model [27], the finite element results were more accurate and effective. The geometrical model did not consider the diameter induction or Poisson’s ratio of the core filament under tension. The mechanical model ignored the interactive relationship between the two components. The maximum negative Poisson’s ratios from the two theoretical models were higher than those obtained on the basis of the experimental results. All these results indicate that the Poisson’s ratio of the core filament and the contact property between the two components needed to be selected in the finite element model. Thus, the finite element model could be used to analyze the deformation behavior and predict the auxetic effect of complex yarns with helical structure.

#### 3.1.2. The Effect of Initial Helical Angle

**Figure 4:**
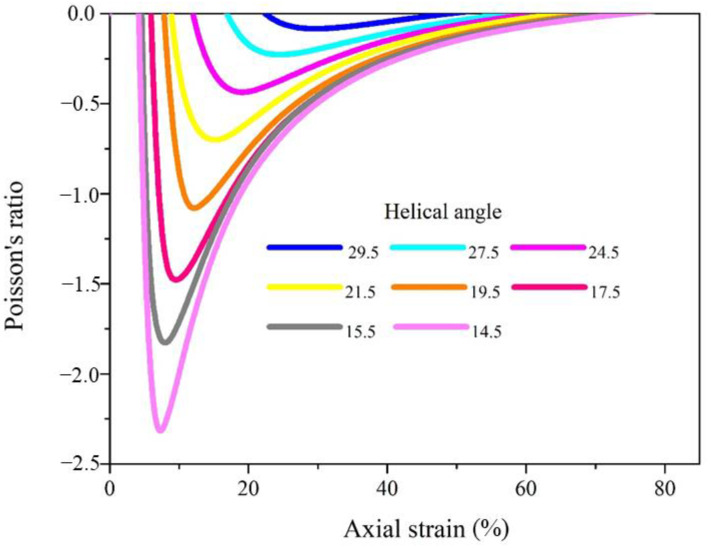
The effect of helical angle on the auxetic property of auxetic yarns.

Finite element method can be used for both prediction and systematic research, and has different limitations than experimental study. As the initial helical angle is the key point of the auxetic behavior, the Poisson’s ratios under the axial strain of complex yarns with helical angles from 14.5° to 29.5° are shown in Figure 4. With lower helix angle, the negativity of the Poisson’s ratio and the expansion effect of complex yarns increased. When the helical angle was larger than 30°, the complex yarns presented a nearly non-auxetic effect. Additionally, complex yarns with lower helical angle presented the most negative Poisson’s ratio under smaller axial strain.

**Figure 5:**
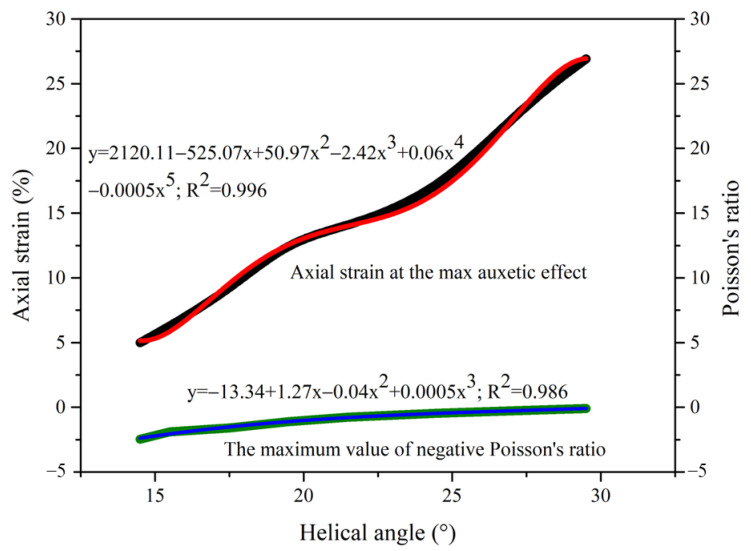
The most negative values of Poisson’s ratio and the related axial strains of auxetic yarns with different helical angles. The fitting results were marked as red and blue lines separately.

As shown in Figure 5, complex yarn with initial helix angle of 14.5° demonstrated the most negative Poisson’s ratio of −2.5 under an axial strain of 5.0%, and yarn with helix angle of 29.5° presented the most negative Poisson’s ratio of −0.1 under an axial strain of 26.9%. The most negative Poisson’s ratio of auxetic yarn and the corresponding axial strain showed nonlinear negative and positive correlations with the initial helix angle, respectively. This was mainly related to the deformation behavior of the two components of complex yarns under a stretching state. When the complex yarn had a smaller helical angle, the core filament went from straight state to helical state, which led to increasing contour diameter and auxetic effect. 

#### 3.1.3. The Effect of Yarn Components’ Poisson’s Ratios

**Figure 6:**
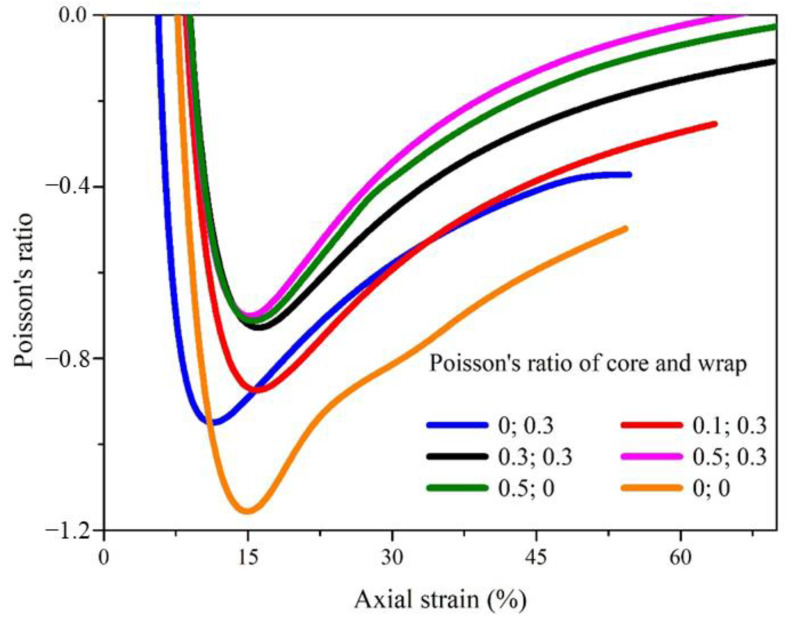
The effect of filament Poisson’s ratio on the auxetic property of complex yarns.

Finite element analysis was used to simulate the effect of the Poisson’s ratios of the two components on the deformation behavior of complex yarns to clarify the material selection requirements for designing and application. As shown in Figure 6, the Poisson’s ratios of complex yarns based on materials with different Poisson’s ratios presented similar variation trends. However, when the Poisson’s ratios of both components were set to zero, the complex yarn had the most negative Poisson’s ratio. These results demonstrate that the two components, especially the core filament, with positive Poisson’s ratio were unfavorable for the auxetic behavior. The main reason was that the diameter of the core filament with positive Poisson’s ratio decreased with increasing axial tension.

The related parameters and results are shown in Table 1. When the Poisson’s ratios of the two components were set to zero, the most negative Poisson’s ratio was −1.2 under an axial strain of 16.5%, which was larger than all other complex yarns. It showed the effect of the deformable characteristic of the core filament on diameter and auxetic property of complex yarns. Thus, both the materials and structural parameters should be considered comprehensively to get the improved designing of helical auxetic yarns.

#### 3.1.4. The Effect of Friction between Contacting Surfaces

**Figure 7:**
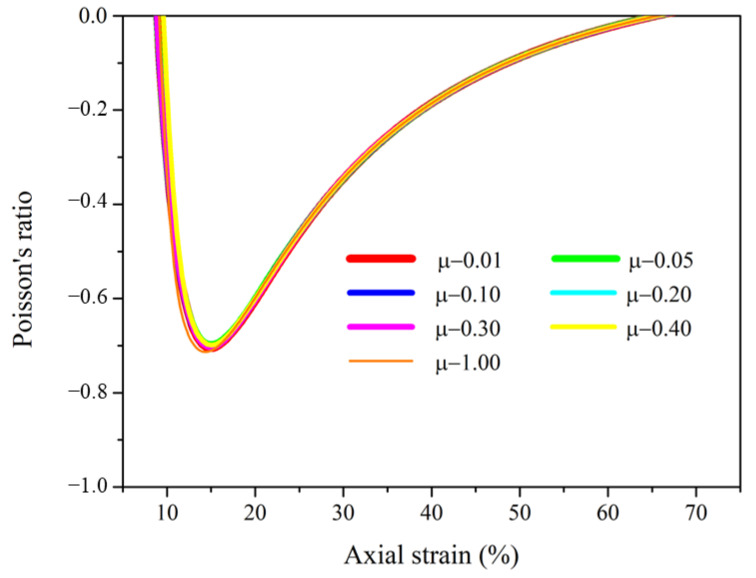
The effect of friction coefficient between yarn components on the Poisson’s ratios of auxetic yarns.

To study the effect of the interaction of the two components on auxetic behavior, finite element models with different friction coefficients μ ranging from 0.01 to 1 were selected and simulated. The Poisson’s ratios with axial strain of the helical auxetic yarns are shown in Figure 7. When the value of friction coefficient μ was 0, the positive Poisson’s ratio of the complex yarn also indicated the indispensable role of the interaction between the two components. However, all the curves presented good consistency, suggesting that the value of friction coefficient between the contacting surfaces had little effect on the auxetic effect of the complex yarns.

### 3.2. Auxetic Property of a Pair of Complex Yarns

**Figure 8:**
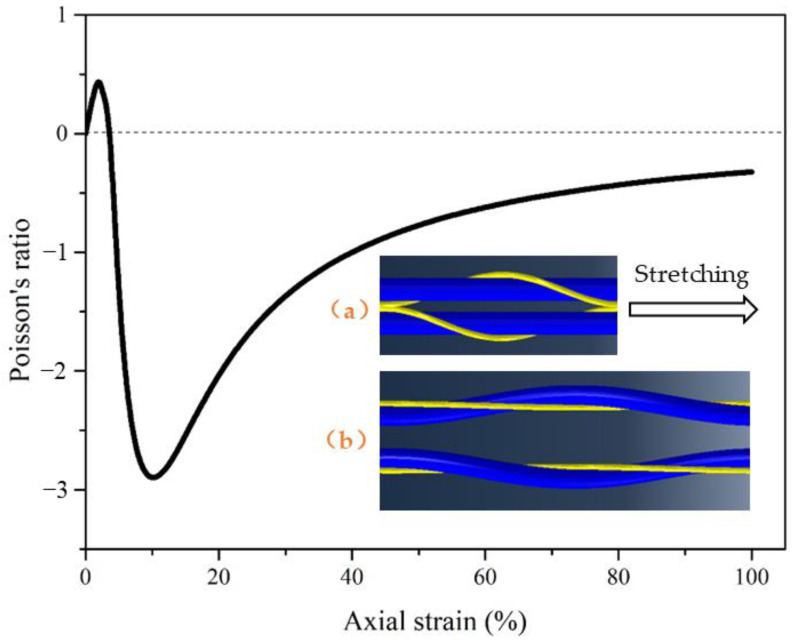
Poisson’s ratio and pore-opening effect of a pair of yarns under axial strain: (**a**) initial state; (**b**) stretching state.

As shown in Figure 8, two groups of auxetic yarns were symmetrically laid together. Under axial tension, there was an obviously increasing pore effect and auxetic behavior. The deformation behavior of the core filaments led to the expansion of the whole filaments. The variation trend was similar with that of the single complex yarn. However, the most negative Poisson’s ratio was nearly −3 under 10% axial strain, which was much more negative than that of the single complex yarn. This was mainly due to the symmetrical arrangement of the two groups of complex yarns. Under axial tension, the core filament deformed outward, leading to the improvement of the contour diameter and expansion effect of the whole yarn. This demonstrates the possibility of obtaining fabrics with higher auxetic effect by the optimization of structural design.

### 3.3. Auxetic Mechanism

**Figure 9:**
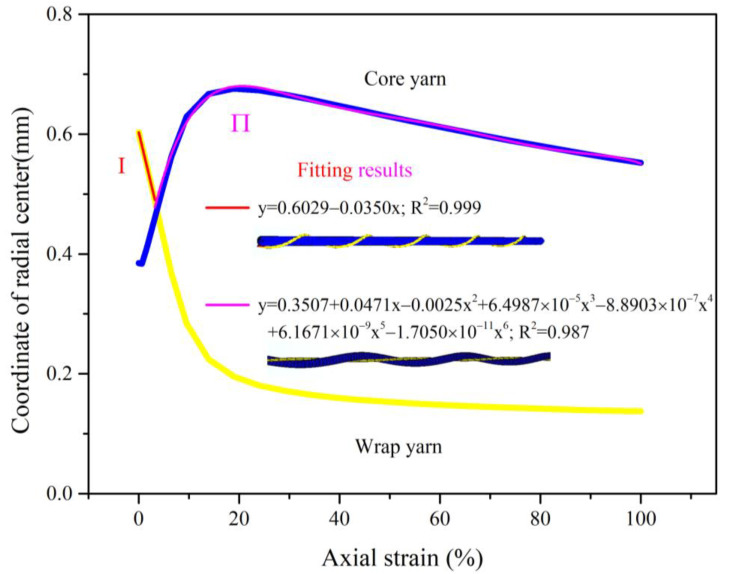
Radial center of auxetic yarn under axial strain.

Under axial tension, the deformation of yarn components made the centerline of the whole complex yarn dynamically change. The coordinates of the highest point and the lowest point along the radial direction of the core filament and the wrap filament were extracted separately, and the average value was used as the central position of complex yarns. As shown in Figure 9, the overall outline of the complex yarn was the wrap filament in the first stage as a result of the helically wrapping structure. With increasing axial strain, the core filament was in a helical state, as was the overall outline of the complex yarn. 

**Figure 10:**
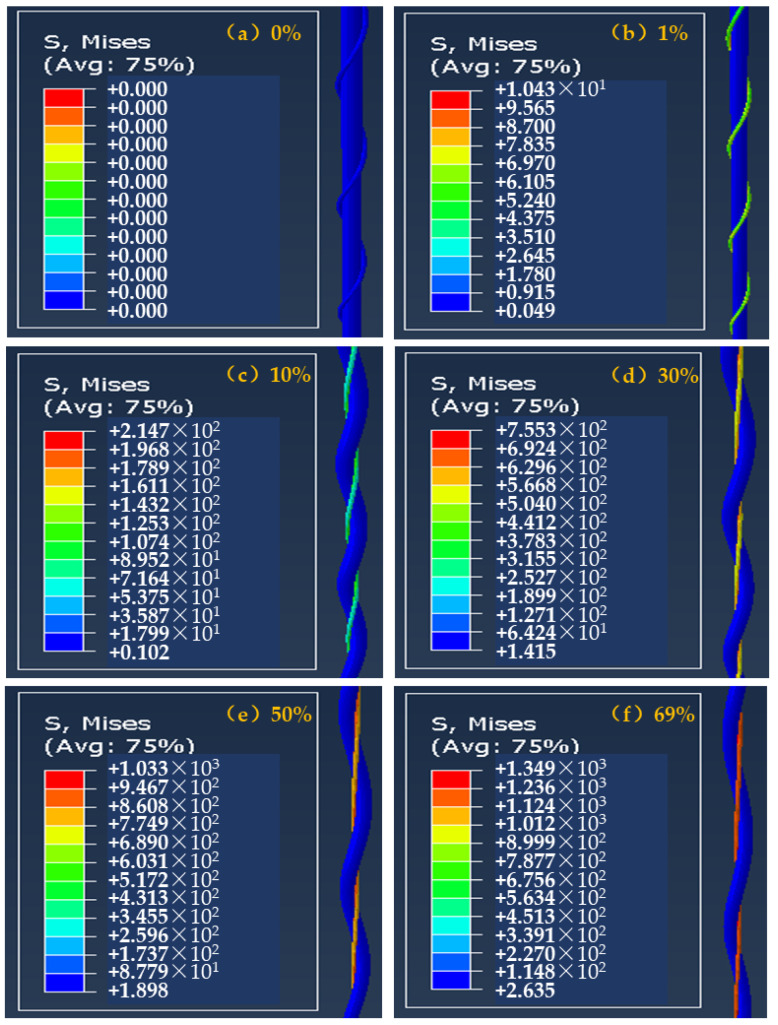
Mises stress distributions at different tensile strains of auxetic yarns: (**a**) 0%; (**b**) 1%; (**c**) 10%; (**d**) 30%; (**e**) 50%; (**f**) 69%.

Obtained from the finite element simulation and analysis, the deformation behavior and stress distribution of helical auxetic yarns under different axial strain are shown in Figure 10. The stress was mainly distributed on the wrap filament under axial tension. With increasing axial strain, the stress on the wrap filament increased significantly. The main reason for this was that the tensile modulus of the wrap filament was much larger than that of the core filament. The wrap filament was gradually strengthened from helical state to horizontal state, which continued until breaking. However, the elastic core filament presented axial elongation at lower stress.

Figure 10a shows the initial state of the complex yarn and the outer outline is the wrap filament. Under lower axial strain, the outer outline was still the wrap filament and the complex yarn showed a positive Poisson’s ratio, as shown in Figure 10b. With increasing axial strain, the wrap filament was gradually straightened from the helical state. However, the helical core filament made the overall contour diameter of complex yarn increase, presenting an auxetic effect. The outer contour of complex yarn was the core, as shown in Figure 10c. With increasing axial strain as shown in Figure 10d–f, the elongation of the two components led to a smaller diameter of the complex yarn and a positive value of the Poisson’s ratio. Thus, the deformation behavior of the core filament and the mechanical property of the wrap filament are key factors for improving the auxetic effect of helical complex yarns.

## 4. Conclusions

We developed and carried out a finite element model and analysis of helical auxetic yarns with two components. Based on the simulation results, the effects of helical angle, interactive relationship and stress distribution on deformation behavior and auxetic property were discussed.

(1) The complex yarn with smaller helical angle showed a greater auxetic effect under smaller axial strain.
(2) The Poisson’s ratios of the components, especially the core filament, affected the auxetic property. This is mainly attributable to the deformable characteristic of the core filament with positive Poisson’s ratio. The interaction between the two components was indispensable for the auxetic effect. However, different friction coefficients between contact surfaces of the two components led to nearly no difference in expansion behavior.
(3) A pair of auxetic yarns being laid together led to a more negative Poisson’s ratio than a single yarn, and showed an obvious pore effect.
(4) During the axial tension process, the stress was always distributed on the stiff wrap filament. The difference of the modulus led to the deformation behavior and auxetic effect. The structure and outside contour of the helical complex yarn varied with the axial strain. This could be a valuable prediction model for radial strain and theoretical Poisson’s ratio by finite element method.

## Figures and Tables

**Table 1:** Auxetic property of complex yarns with filaments with different Poisson’s ratios.

Poisson’s Ratio of Core	Poisson’s Ratio of Wrap	Most Negative Poisson’s Ratio	Related Axial Strain (%)
0	0.3	−1.0	9.9
0.1	0.3	−0.9	13.5
0.3	0.3	−0.8	14.6
0.5	0.3	−0.8	14.0
0.5	0	−0.8	15.4
0	0	−1.2	16.5

## Data Availability

Not applicable.
